# Hydrocephalus Induced by Intraventricular Peroxiredoxin-2: The Role of Macrophages in the Choroid Plexus

**DOI:** 10.3390/biom11050654

**Published:** 2021-04-29

**Authors:** Ting Chen, Xiaoxiao Tan, Fan Xia, Ya Hua, Richard F. Keep, Guohua Xi

**Affiliations:** 1 Department of Neurosurgery, University of Michigan, Ann Arbor, MI 48109, USA; ice1009@zju.edu.cn (T.C.); 13777843135@139.com (X.T.); fxia@umich.edu (F.X.); yahua@umich.edu (Y.H.); rkeep@umich.edu (R.F.K.); 2 School of Medicine, Zhejiang University, Hangzhou 310027, China

**Keywords:** choroid plexus, dendritic cells, hydrocephalus, inflammation, macrophages, OX-6, Peroxiredoxin 2

## Abstract

The choroid plexus (CP) is the primary source of cerebrospinal fluid in the central nervous system. Recent evidence indicates that inflammatory pathways at the CP may be involved in hydrocephalus development. Peroxiredoxin 2 (Prx2) is a major component of red blood cells. Extracellular Prx2 is proinflammatory, and its release after red blood cell lysis may contribute to hydrocephalus after intraventricular hemorrhage. This study aimed to identify alterations in CP macrophages and dendritic cells following intracerebroventricular Prx2 injection and investigate the relationship between macrophages/dendritic cells and hydrocephalus. There were two parts to this study. In the first part, adult male Sprague–Dawley rats received an intracerebroventricular injection of Prx2 or saline. In the second part, Prx2 was co-injected with clodronate liposomes or control liposomes. All animals were euthanized at 24 h after magnetic resonance imaging. Immunohistochemistry was used to evaluate macrophages in CP, magnetic resonance imaging to quantify hydrocephalus, and histology to assess ventricular wall damage. The intracerebroventricular injection of Prx2 not only increased the OX-6 positive cells, but it also altered their location in the CP and immunophenotype. Co-injecting clodronate liposomes with Prx2 decreased the number of macrophages and simultaneously attenuated Prx2-induced hydrocephalus and ventricular wall damage. These results suggest that CP macrophages play an essential role in CP inflammation-induced hydrocephalus. These macrophages may be a potential therapeutic target in post-hemorrhagic hydrocephalus.

## 1. Introduction

The choroid plexuses (CPs) are the primary source of cerebrospinal fluid (CSF), and they play an essential role in maintaining central nervous system (CNS) homeostasis. CPs consist of a monolayer of cuboidal epithelial cells surrounding a stromal core of capillaries, as well as various immune cells including stromal macrophages, Kolmer’s epiplexus cells and dendritic cells (DCs) [1,2]. Prior research has uncovered that activation of inflammatory pathways at the CP can induce CSF hypersecretion and result in hydrocephalus after intraventricular hemorrhage [3]. However, alterations in CP immune cells under inflammatory conditions and the relationship between immune cells and hydrocephalus have yet to be elucidated.

Peroxiredoxin 2 (Prx2) is a major component of red blood cells (RBCs) [4]. It can be released into extracellular space after hemolysis and evoke a series of severe inflammatory responses [5]. Our previous studies revealed that ionized calcium-binding adaptor molecule 1 (Iba-1, a macrophage/microglia marker) positive cells were increased at the CP after subarachnoid hemorrhage and in blood component (thrombin and Prx2)-induced hydrocephalus [6,7]. This suggests that CP resident macrophages may play a role in hydrocephalus development. 

Liposomes are artificially prepared lipid vesicles and can be used as carriers to encapsulate hydrophilic drug molecules such as clodronate. Clodronate liposomes can be recognized and engulfed by macrophages and can subsequently induce cell death [8]. Selectively depleting macrophages by clodronate liposomes has been investigated in neurological diseases, such as hypertension-induced cognitive dysfunction and intracerebral hemorrhage [9,10]. However, the role of depleting macrophages in hydrocephalus has not been studied.

The present study, therefore, aimed to determine: (a) the activation of OX-6 (major histocompatibility complex II) positive cells on CP following the intracerebroventricular (icv) injection of Prx2; and (b) the effects of clodronate liposomes on the hydrocephalus development induced by Prx2.

## 2. Materials and Methods

### 2.1. Animal Preparation and Intracerebroventricular Injection

Animal protocols were approved by the University of Michigan Committee on the Use and Care of Animals. The University of Michigan has an Animal Welfare Assurance on file with the Office for Protection from Research Risks and is fully accredited by the American Association for the Accreditation of Laboratory Animal Care. The studies followed the Guide for the Care and Use of Laboratory Animals (National Research Council) and comply with the ARRIVE guidelines for reporting in vivo experiments [11].

A total of 28 Sprague–Dawley (SD) male rats (aged 3–4 months; weighing 250–350 g) were used in this study (Charles River Laboratories, Portage, MI, USA). Animals were anesthetized with pentobarbital (50 mg/kg, i.p.). The core body temperature was maintained at 37.5 °C with a feedback-controlled heating pad. Rats were then positioned in a stereotaxic frame (Kopf Instruments, Tujunga, CA, USA). A cranial burr hole (1 mm) was drilled 0.6 mm posterior, 4.5 mm ventral, and 1.7 mm lateral to the bregma, and a 26-gauge needle was inserted perpendicularly through the burr hole into the right lateral ventricle.

A micro-infusion pump was used to inject 50 μL of saline and recombinant Prx2 solution (1 mg/mL; Novus Biological Corp, Centennial, CO, USA; NBP2-52150, 25 μL, diluted with an equal volume of saline). Prx2 solution was also co-injected with clodronate liposome (7 mg/mL; FormuMax Scientific, Inc., Sunnyvale, CA, USA; F70101C-N, 25 μL) or control liposome (7 mg/mL; FormuMax Scientific, Inc.; F70101-N, 25 μL). The needle was kept in place for 10 min to prevent backflow and then withdrawn. Bone wax was used to fill the hole, and the skin incision was subsequently sutured closed.

### 2.2. Experimental Groups

There were two parts to this study. First, 12 male rats were randomly divided into Prx2 and saline groups (n = 6 in each group). They received a 50-μL icv injection of either Prx2 or saline and were euthanized one day after magnetic resonance imaging (MRI). Brains were harvested for histology. Second, 14 male rats were randomly divided into two groups (n = 7 in each group) with icv Prx2 co-injected with either clodronate or control liposomes. All rats had MRI scans before euthanasia one day after injection, and their brains were harvested for histology. Randomization was performed using odd/even numbers for the treatment groups. Dead animals were excluded from this study.

### 2.3. Immunohistochemistry and Immunofluorescence Double Staining

All rats were euthanized with pentobarbital (390 mg/kg, intraperitoneally) and then underwent transcardiac perfusion with 4% paraformaldehyde in 0.1 mol/L PBS (pH 7.4). Brains were removed, kept in 4% paraformaldehyde for 24 h, and then immersed in 30% sucrose for two to three days at 4 °C. Brains were embedded in optimal cutting temperature compound (Sakura Finetek, Torrance, CA, USA) and sectioned coronally (18-μm-thick slices). Immunohistochemical staining was performed using the avidin-biotin complex technique as previously described [12]. 

The primary antibodies used in this study were mouse anti-rat OX-6 (MHC Class II RT1B antibody, 1:200 dilution; Bio-Rad Laboratories, Hercules, CA, USA; MCA46R) and rabbit anti-MPO (myeloperoxidase; 1:300 dilution; Invitrogen, Waltham, MA, USA; PA5-16672). For double immunofluorescence staining, the primary antibodies were the same anti-OX-6 antibody and goat anti–Iba-1 (anti-ionized calcium-binding adaptor molecule 1, 1:400 dilution; Abcam, Cambridge, MA, USA, ab5076). The secondary antibodies were Alexa Fluro 488-conjugated donkey anti-goat mAb (1:500 dilution, Invitrogen) and Alexa Fluro 594-conjugated donkey anti-mouse mAb (1:500 dilution, Invitrogen). Negative controls excluded the primary antibodies.

### 2.4. Magnetic Resonance Imaging and Ventricular Volume Measurement

MRIs were performed with rats anesthetized by 2% isoflurane using a 9.4-T Varian MR scanner (Varian, Palo Alto, CA, USA) with a T2 fast spin-echo sequence. A total of 25 coronal slices were obtained for each scan with a view field of 35 × 35 mm^2^ and a slice thickness of 0.5 mm. Ventricular volumes were measured as described previously [12]. Bilateral ventricles were outlined in each slice and measured by another person blinded to the experiments. The ventricular volume was calculated by multiplying the area measured in each slice with the slice thickness and then summing up all the slices.

### 2.5. Ventricular Wall Damage Measurements and Cell Counting

Analyses of ventricular wall damage were performed as described previously [13]. Hematoxylin and eosin-stained brain sections were obtained for each rat. The length of the ependyma that was disrupted or detached from the periventricular parenchyma was measured, as well as the total bilateral ventricular wall length. The degree of ventricular wall damage was calculated as a percentage by dividing the length of disruption over the total ventricular surface perimeter. Cell counting was based on coronal brain sections (0.5~1.5 mm posterior to Bregma) in high-power images (×40 magnification). Three views were taken from both the left and right lateral ventricle CP to maximally cover the whole CP in a brain section. To analyze inflammatory cells’ changes at the CP in immunohistochemical staining, the number of positive cells was calculated as a percentage of the total CP epithelial cell count of the bilateral CPs for each rat. In immunofluorescence staining, the number of positive cells was calculated as a percentage of the total DAPI count. All analyses were repeated three times by a blinded investigator using Image J.

### 2.6. Statistical Analysis

Values are shown as means ± SD. A Student *t*-test and one-way ANOVA with a Tukey post hoc test were used for data analyses. Differences were considered significant with *p* < 0.05.

## 3. Results

### 3.1. OX-6 Positive Cells Were Increased after Prx2 Injection

**Figure 1:**
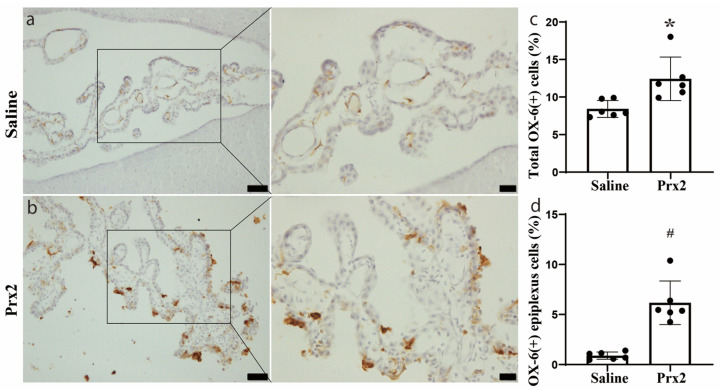
Peroxiredoxin 2 (Prx2) alters the number and location of major histocompatibility complex class II (OX-6) positive (+) cells at the choroid plexus (CP). (**a**) Examples of OX-6 immunohistochemistry at the choroid plexus one day after intracerebroventricular (icv) saline injection; (**b**) Examples of OX-6 immunohistochemistry at the choroid plexus one day after icv Prx2 injection. Note the intense staining of apical epiplexus cells after Prx2 injection; (**c**) The numbers of OX-6 positive cells in saline- and Prx2-injected animals were quantified and expressed as a % of all CP cells; (**d**) The numbers of OX-6 positive epiplexus cells in the two groups were also analyzed. Values are means; n = 6; * *p* < 0.05 vs. saline group; # *p* < 0.01 vs. saline group. Scale bar = 50 μm at low magnification, 20 μm at high magnification. Dots represent data for each animal.

In the current study, OX-6 positive (OX-6 (+)) cells were detected in the CP in the saline control group, with most of them located in the CP stroma (Figure 1a). This result is consistent with previous studies [14,15]. One day after icv injection of Prx2, the number, morphology, and location of OX-6 (+) cells at the CP were altered. In addition, some OX-6 (+) cells were free-floating in the CSF (Figure 1b). The number of CP OX-6 (+) cells was significantly increased after Prx2 injection (12.4 ± 2.9 versus 8.4 ± 1.1% of all CP cells in the saline group; *p* < 0.05; Figure 1c). The number of OX-6 (+) epiplexus cells (CP apical surface) was also significantly increased (6.2 ± 2.2 versus 0.9 ± 0.4% of all CP cells in the saline group; *p* < 0.01; Figure 1d).

### 3.2. Immunophenotypic Changes of CP OX-6 Positive Cells after Prx2 Injection

**Figure 2:**
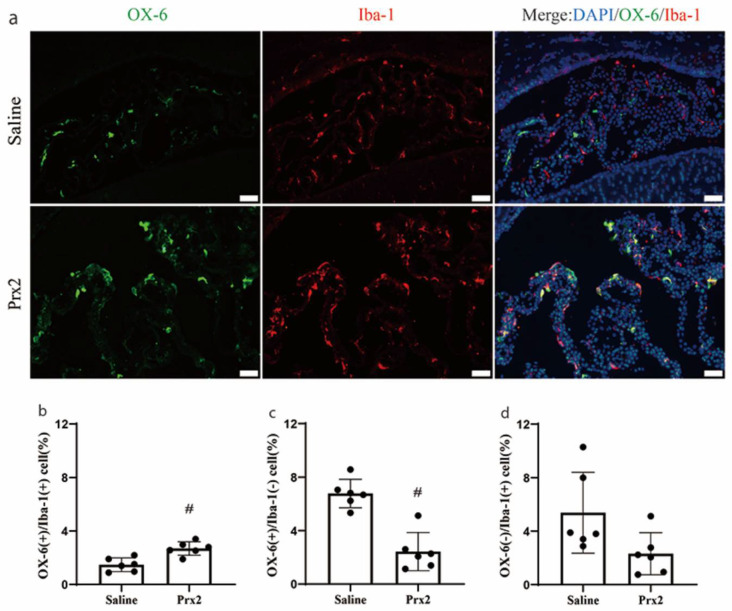
Prx2-induced alterations in the immunophenotype of OX-6 positive (+) cells and Ionized calcium-binding adaptor molecule 1 (Iba-1) (+) cells at the choroid plexus. (**a**) Examples of double labeling of OX-6 and Iba-1 at the choroid plexus one day after icv saline and Prx2 injections. (**b**–**d**) The number of OX-6(+)/Iba-1(+) cells, OX-6(+)/Iba-1(−) cells (defined as dendritic cells) and OX-6(−)/Iba-1(+) cells were quantified as a percentage of all choroid plexus cells. Values are the mean ± SD; n = 6; # *p* < 0.01 vs. saline group. Scale bar = 50 μm. Dots represent data for each animal.

There was a shift in the phenotype of CP immune cells after Prx2 injection. In saline-injected rats, most OX-6 (+) cells were located in the CP stroma, while most Iba-1(+) cells were epiplexus cells. After Prx2 injection, the number of double positive, OX-6(+)/Iba-1(+), cells significantly increased (2.7 ± 0.5 vs. 1.5 ± 0.5% of all CP cells in the saline group; *p* < 0.01; Figure 2a,b). After Prx2 injection, the number of OX-6(+)/Iba-1(−) cells decreased (2.4 ± 1.4 vs. 6.8 ± 1.0% of all CP cells with saline; *p* < 0.01; Figure 2c). A prior investigation defined OX-6(+)/Iba-1(−) cells at the CP as dendritic cells [16]. There was a nonsignificant trend towards a reduction in the number of OX-6(−)/Iba-1(+) cells with Prx2 injection (2.3 ± 1.6 vs. 5.4 ± 3.0% in the saline group; *p* > 0.05; Figure 2d).

### 3.3. CP Macrophages Were Depleted by Intracerebroventricular Clodronate Liposome

**Figure 3:**
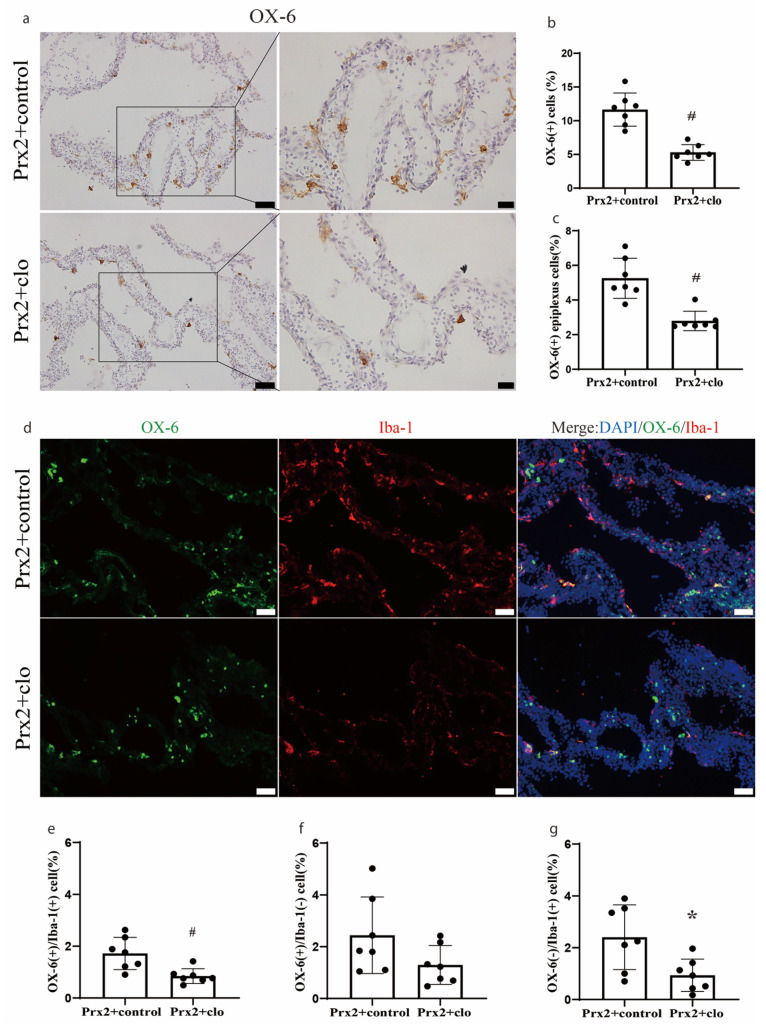
Effect of clodronate liposomes on the number and type of immune cells at the choroid plexus one day after icv injection of Prx2. (**a**) Examples of OX-6 immunohistochemistry after co-injection of Prx2 + clodronate liposomes (Prx2 + clo) or Prx2 + control liposomes (Prx2 + control). (**b**) The numbers of OX-6(+) cells were quantified, and (**c**) the OX-6 positive (+) epiplexus cells on the choroid plexus were analyzed. (**d**) Examples of the double immunofluorescence labeling of OX-6 and Iba-1 at the choroid plexus one day after icv Prx2 co-injected with clodronate or control liposomes. (**e**–**g**) The numbers of OX-6(+)/Iba-1(+), OX-6(+)/Iba-1(−) and OX-6(−)/Iba-1(+) cells were quantified as a % of all choroid plexus cells. Values are the mean ± SD; n = 7; # *p* < 0.01 vs. control group. * *p* < 0.05 vs. control group. Scale bar = 50 μm at low magnification and immunofluorescence figure, 20 μm at high magnification. Dots represent data for each animal.

The number of OX-6(+) cells was significantly decreased if Prx2 was co-injected with clodronate liposomes (5.3 ± 1.2% of all CP cells) compared to co-injection with control liposomes (12.2 ± 2.2%; *p* < 0.01; Figure 3a,b). The number of OX-6(+) epiplexus cells located on the CP apical surface was also significantly decreased with clodronate liposomes (2.8 ± 0.6 vs. 5.5 ± 1.0% with control liposomes; *p* < 0.01; Figure 3c). The effect of clodronate liposomes was further examined by double immunofluorescence staining (Figure 3d). Clodronate liposomes significantly depleted the OX-6(+)/Iba-1(+) cells (0.8 ± 0.3 vs. 1.7 ± 0.7% with control liposomes; *p* < 0.01; Figure 3e) and OX-6(−)/Iba-1(+) cells (0.9 ± 0.6 vs. 2.4 ± 1.2% with control liposomes; *p* < 0.05; Figure 3g). There was no significant difference in OX-6(+)/Iba-1(−) cells with clodronate liposomes (1.3 ± 0.7 vs. 2.4 ± 1.5% in the control group; Figure 3f).

### 3.4. Macrophage Depletion Attenuated Neutrophil Infiltration in CP

**Figure 4:**
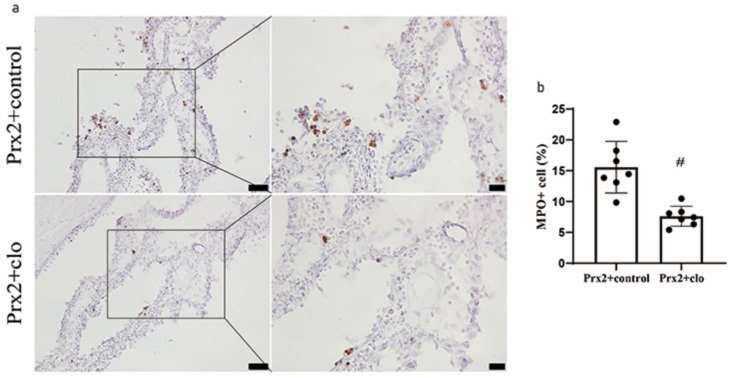
Effect of clodronate liposomes on Prx2-induced neutrophil infiltration. (**a**) Examples of myeloperoxidase (MPO) immunohistochemistry at the choroid plexus one day after icv Prx2 + clodronate liposome (Prx2 + clo) and Prx2 + control liposome (Prx2 + control) injections. (**b**) The numbers of MPO positive cells were quantified as a % of all choroid plexus cells. Values are the mean ± SD; n = 7; # *p* < 0.01 vs. control group. Scale bar = 50 μm at low magnification, 20 μm at high magnification. Dots represent data for each animal.

Neutrophil infiltration is correlated with inflammatory severity, and the number of myeloperoxidase (MPO, neutrophil marker) positive cells increased in CP after Prx2 injection in our previous study [7]. When Prx2 was co-injected with clodronate liposomes, the number of MPO+ cells significantly decreased (7.6 ± 1.6% of all CP cells) compared to control liposomes (16.5 ± 3.7%; *p* < 0.01; Figure 4a,b).

### 3.5. Macrophage Depletion Attenuated Hydrocephalus and Ventricular Wall Damage

**Figure 5:**
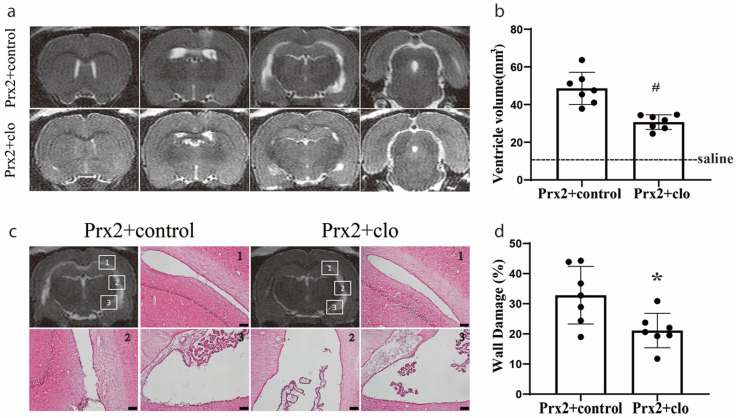
Injection of clodronate liposomes attenuated Prx2-induced hydrocephalus and ventricular wall damage. (**a**) Examples of T2 magnetic resonance imaging (MRI) in rats one day after injection of Prx2 + control liposomes (Prx2 + control) and Prx2 + clodronate liposomes (Prx2 + clo) into the right lateral ventricle. (**b**) Quantification of the ventricular volume in the two groups. Values are the mean ± SD; n = 7; # *p* < 0.01 vs. control group. (**c**) Examples of hematoxylin and eosin (HE)-stained sections showing ventricular wall damage one day after icv injection of Prx2 + control liposomes and Prx2 + clodronate liposomes. (**d**) The percentage of ventricle wall that was damaged is quantified in the bar graph. Values are the mean ± SD; n = 7; * *p* < 0.05 vs. control groups. Scale bar = 100 μm. Dots represent data for each animal.

We have previously found that the icv injection of Prx2 causes hydrocephalus and ventricular wall damage [7]. In the current study, Prx2 co-injected with clodronate liposomes significantly reduced the ventricular dilation induced by Prx2 (30.7 ± 3.9 mm^3^) compared with control liposomes (48.1 ± 9.3 mm^3^) at one day (*p* < 0.01; Figure 5a,b). Compared to rats in the control liposome group, a significant reduction in ventricular wall damage was also found in rats with Prx2 + clodronate liposome co-injection (21 ± 5 vs. 33 ± 9% in the control liposome group; *p* < 0.05; Figure 5c,d).

## 4. Discussion

The main findings of this study were: (a) OX-6 (+) cells are predominately located in the stroma of CP in control rats; (b) in contrast, most of them appeared on the CP apical surface after Prx2 injection with an increased number; (c) the OX-6(+) cells in CP were reduced when Prx2 was co-injected with clodronate liposomes; (d) co-injecting Prx2 with clodronate liposomes attenuated neutrophil infiltration, hydrocephalus and ventricular wall damage.

The CP is considered as a niche for various immune cells in the CNS. Three major types of resident immune cells in CP have been described [1]. (1) Epiplexus macrophages, also known as Kolmer’s cells, are localized at the apical side of CP epithelial cells. This group of cells faces the CSF space and are thought to function as local antigen-presenting cells (APCs) [17], which can be detected by ED2 (CD163) and Iba-1 immunohistochemistry staining [6,14]. (2) CP macrophages, which reside in the CP stroma, have a similar immunophenotype with epiplexus macrophages but are normally present in smaller numbers [14]. (3) Dendritic cells (DCs), which primarily locate in the stroma of CP, are MHC II (+)/ED2 (−) dendriform cells and greatly outnumber the CP macrophages [14]. In the current study, we found that most of the OX-6(+) cells in the CP stroma of saline-injected rats were DCs.

The current study examined the effects of the effects of Prx2 on those CP immune cells because (a) Prx2 is a major intracellular component of RBCs and may be released when RBCs lyse after a cerebral hemorrhage, (b) Prx2 is proinflammatory and (c) evidence indicates that icv Prx2 can induce ventriculomegaly [7]. In Prx2-injected rats, the number of OX-6(+) cells increased and there was a shift to the apical side of the CP epithelium. There are three potential possibilities for this phenomenon. The first hypothesis is DC migration. It has been accepted that DCs can migrate from inflammatory tissue to the lymph node through the lymph vessel as antigen-presenting cells. The DCs in CNS can also stimulate autoreactive T cells by in situ presentation of autoantigens [18]. Thus, it is entirely possible for DCs to migrate through the CP epithelium. The second possibility is epiplexus macrophage activation. Macrophages can be activated by inflammatory stimulation and have elevated expression levels of MHC II [19]. The cells detected by OX-6 may include epiplexus macrophages. Distinguishing DCs from other macrophages has proven difficult as these cell populations share certain surface markers. The expression of these markers may undergo real-time variations owing to environmental influences such as inflammatory stimuli [18]. The third possibility is that activated T cells express MHC II [20]. There are no T cells that reside on CP in healthy conditions, but it has been reported that the CP may be a key cerebral invasion route for T cells after stroke [21]. The CP can also promote T cell trafficking as a response to inflammation that impacts adaptive immunity in the CNS [22]. Thus, we suspect that smaller OX-6(+) cells may be activated T cells. 

In this study, we divided the CP immune cells into three groups after Prx2 injection. (1) OX-6(+)/Iba-1(+) cells, which are considered to be activated macrophages, included epiplexus macrophages and stromal macrophages. (2) OX-6(+)/Iba-1(−) cells, which are considered to be DCs but may also contain other antigen-presenting cells such as activated T cells. (3) OX-6(−)/Iba-1(+) cells, which may be some macrophages that do not act as APCs and do not express MHC II. When Prx2 was co-injected with clodronate liposome, the number of OX-6(+)/Iba-1(+) cells and OX-6(−)/Iba-1(+) cells significantly decreased, but that of OX-6(+)/Iba-1(−) cells did not. This result indicates that clodronate liposome can selectively deplete macrophages in CP.

Macrophages and neutrophils play an essential role in inflammatory responses. Macrophages are activated by inflammatory stimulation and produce various proinflammatory cytokines such as interleukin-1β (IL-1β), IL-6, IL-12, IL-23, IL-10, and tumor necrosis factor-α [23,24]. Proinflammatory cytokines, especially IL-1β, have emerged as a powerful driving force for leukocyte recruitment to the CNS [25]. Perivascular macrophages support the recruitment of granulocytes via the release of vascular endothelial growth factor, which increased vascular permeability in a rat model of middle cerebral artery occlusion [26]. Neutrophils produced matrix metalloprotease 9 (MMP-9) and contribute to blood vessel disruption, blood-brain barrier breakdown, and microglial/macrophage responses, while neutrophil depletion reduced infiltration of activated macrophages [27]. Thus, there is a synergistic effect between macrophages and neutrophils in the inflammatory response, which can be interrupted by depleting either of them. This evidence may explain how macrophage depletion attenuated the neutrophil infiltration in our study. 

Recent evidence indicates that the activation of inflammatory pathways at the CP can induce CSF hypersecretion and that this may contribute to hydrocephalus after intraventricular hemorrhage [3]. The depletion of macrophages that adhere to the CP epithelium attenuates neutrophil infiltration and CP inflammation. This suggests that it may reduce secretion of CSF by the CP epithelium, as well as ventricle dilation and wall damage. This needs investigation.

There are several limitations to the current study. It is a proof-of-concept study that determined alterations in CP macrophages and dendritic cells following intraventricular injection of Prx2 and the inflammatory reaction mediated by macrophages in the Prx2-induced hydrocephalus. Further studies are needed to examine the effects of other RBC components which could be potent inflammatory inducers. In addition, it is uncertain whether the nonspecific uptake of clodronate liposomes by other MHC II-positive cells will have an impact on hydrocephalus formation and the inflammatory response. This still needs to be explored in the future.

In summary, resident CP immune cells play an essential role in Prx2-induced inflammation, and they may have similar effects after intraventricular hemorrhage or subarachnoid hemorrhage given the high concentration of Prx2 in RBCs. Depleting the CP macrophages by clodronate liposome attenuates ventriculomegaly by inhibiting the inflammation at the CP. Thus, CP immune cells may be a potential therapeutic target in cerebral hemorrhage-induced hydrocephalus.

## Data Availability

All relevant data are within the paper.

## Abbreviations

(+)	positive
(−)	negative
APCs	Antigen-presenting cells
CNS	Central nervous system
CPs	Choroid plexuses
DCs	Dendritic cells
Iba-1	Ionized calcium-binding adaptor molecule 1
Icv	Intracerebroventricular
MRI	Magnetic resonance imaging
MHC II	Major histocompatibility complex II
MPO	Myeloperoxidase
Prx2	Peroxiredoxin 2
RBCs	Red blood cells
